# Toxicity of amantadine hydrochloride on cultured bovine cornea endothelial cells

**DOI:** 10.1038/s41598-021-98005-9

**Published:** 2021-09-16

**Authors:** Po-Yen Lee, Yu-Hung Lai, Po-Len Liu, Ching-Chih Liu, Chia-Cheng Su, Fang-Yen Chiu, Wei-Chung Cheng, Shiuh-Liang Hsu, Kai-Chun Cheng, Li-Yi Chiu, Tzu-En Kao, Chia-Ching Lin, Yo-Chen Chang, Shu-Chi Wang, Chia-Yang Li

**Affiliations:** 1 Graduate Institute of Medicine, College of Medicine, Kaohsiung Medical University, Kaohsiung, 80708 Taiwan; 2 Department of Ophthalmology, Kaohsiung Medical University Hospital, Kaohsiung Medical University, Kaohsiung, 80708 Taiwan; 3 Department of Ophthalmology, School of Medicine, College of Medicine, Kaohsiung Medical University, Kaohsiung, 80708 Taiwan; 4 Department of Respiratory Therapy, College of Medicine, Kaohsiung Medical University, Kaohsiung, 80708 Taiwan; 5 Department of Ophthalmology, Chi Mei Medical Center, Tainan, 71004 Taiwan; 6 Division of Urology, Department of Surgery, Chi-Mei Medical Center, Tainan, 71004 Taiwan; 7 Department of Senior Citizen Service Management, Chia Nan University of Pharmacy and Science, Tainan, 71710 Taiwan; 8 Graduate Institute of Biomedical Sciences, and Research Center for Tumor Medical Science, and Drug Development Center, China Medical University, Taichung, 40402 Taiwan; 9 Department of Ophthalmology, Kaohsiung Municipal Siaogang Hospital, Kaohsiung, 81267 Taiwan; 10 Department of Ophthalmology, Kaohsiung Municipal Ta-Tung Hospital, Kaohsiung, 80145 Taiwan; 11 Department of Medical Laboratory Science and Biotechnology, Kaohsiung Medical University, Kaohsiung, 80708 Taiwan; 12 Center for Cancer Research, Kaohsiung Medical University, Kaohsiung, 80708 Taiwan

**Keywords:** Eye diseases, Pharmacology, Eye manifestations, Chemical safety

## Abstract

Amantadine hydrochloride (HCl) is commonly prescribed for treating influenza A virus infection and Parkinson’s disease. Recently, several studies have indicated that the use of amantadine HCl is associated with corneal edema; however, the cytotoxic effect of amantadine HCl has not been investigated. In the present study, the effects of amantadine HCl on cell growth, proliferation, and apoptosis in bovine cornea endothelial cells, and in vitro endothelial permeability were examined. Results showed that lower doses of amantadine HCl do not affect cell growth (≤ 20 μΜ), whereas higher doses of amantadine HCl inhibits cell growth (≥ 50 μΜ), induces apoptosis (2000 μΜ), increases sub-G1 phase growth arrest (2000 μΜ), causes DNA damage (≥ 1000 μΜ), and induces endothelial hyperpermeability (≥ 1000 μΜ) in bovine cornea endothelial cells; additionally, we also found that amantadine HCl attenuates the proliferation (≥ 200 μΜ) and arrests cell cycle at G1 phase (≥ 200 μΜ) in bovine cornea endothelial cells. In the present study, we measured the cytotoxic doses of amantadine HCl on cornea endothelial cells, which might be applied in evaluating the association of corneal edema.

## Introduction

Amantadine hydrochloride (HCl) is a prophylactic agent originally approved in October 1966 by the Food and Drug Administration (FDA) specifically against Asian influenza^1^. Subsequently, Schwab et al. demonstrated that amantadine HCl was useful for treating Parkinson’s disease (PD) as monotherapy^2^ and in combination therapy with L-dopa and anticholinergic drugs^3^. In April 1973, the FDA approved the use of amantadine HCl for alleviating symptoms of PD, and it has been widely used in the treatment of PD since then^4^. In addition, amantadine HCl also shows beneficial effects in the treatment of dengue virus infection^5^, inhibition of West Nile Virus multiplication^6^, and in off-label use to treat fatigue in multiple sclerosis^7^.

Amantadine HCl has been demonstrated to inhibit influenza A virus replication through inhibition of the ion channel function of M2 protein^8^. In vitro doses from 1 to 10 µM are enough to achieve 50% inhibition of most influenza viruses^8^. For the treatment of PD, amantadine HCl acts as a weak antagonist of the NMDA receptor that increases dopamine release and blocks dopamine reuptake^9^. The recommended dosage of amantadine HCl in adults is 200 mg daily, and the blood plasma values are in the range of 0.3–0.6 μg/mL^10,11^, equal to 1.59–3.19 μM. Kornhuber et al. indicated that mean amantadine HCl concentrations in brain tissue ranged from 48.2 to 386 μM when patients received amantadine HCl for ≥ 10 days and had a drug-free time of ≤ 3 days^12^, whereas the amantadine HCl concentrations in cerebrospinal fluid and serum were in the low micromolar range (< 17 μM)^12^. In addition, the serum levels of amantadine HCl ranged between 2.6 (a patient who received 200 mg just for 1 day) and 16.3 μM (a patient who received 600 mg amantadine HCl for 10 days)^12^. These results indicate that amantadine HCl concentration is distributed across a wide range in different tissues. Dosage, duration of treatment, and drug-free time are all associated with mean amantadine HCl concentration. Although the mean amantadine concentration in the cornea has not yet been examined, it has been reported that amantadine has high penetrative activity into the brain after infusion in rats (brain concentration of amantadine was 16-fold higher than free concentration in serum)^13^.

In recent years, an increasing number of case reports have indicated that the use of amantadine HCl is associated with corneal edema^14–22^. A nationwide cohort study in Taiwan also demonstrated that amantadine HCl increases the risk of corneal edema in a dose-dependent manner^11^; however, how this occurs is still unclear. Corneal endothelium controls the water content of the corneal stroma, whereas corneal endothelial decompensation leads to overhydration of the cornea, known as corneal edema^23^. Thus, in the present study, we aimed to examine whether amantadine HCl affects cell growth, proliferation and apoptosis in bovine cornea endothelial cells.

## Results

### Lower doses of amantadine HCl (≤ 20 μM) do not affect cell growth and viability in bovine cornea endothelial cells

**Figure 1:**
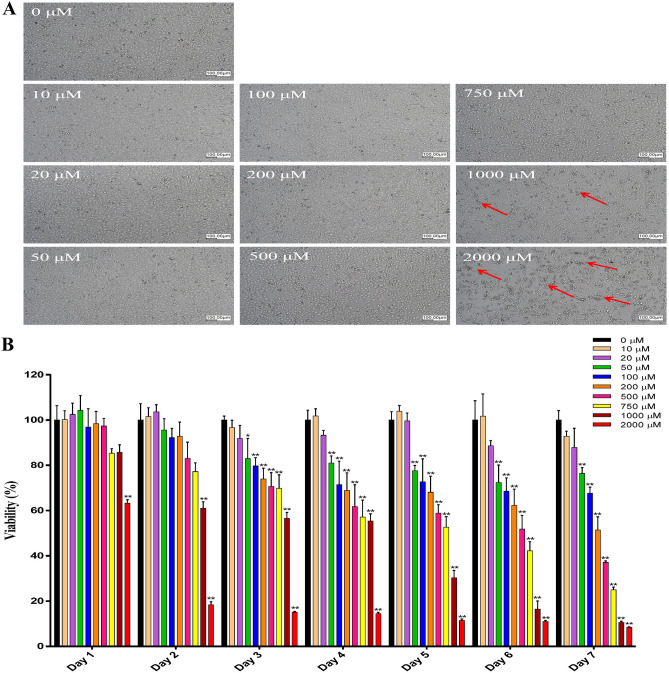
Effect of amantadine HCl on cell growth of bovine cornea endothelial cells. (**A**) BCE C/D-1b cells were treated with various doses of amantadine HCl (0–2000 μM) for 24 h. Cell morphology was monitored by phase-contrast microscopy. The red arrow indicates dead cells that became detached and floated after treatment. Assays were carried out in triplicates, and the results are representative of three independent experiments. (**B**) BCE C/D-1b cells were treated with various concentrations of amantadine HCl (0–2000 μM) for 1–7 days. Cell viability was examined by MTT assay. The data are presented as means ± SD of three independent experiments. Statistical significance was represented as follows: *p < 0.05 or **p < 0.01 vs. untreated control.

To examine the cytotoxicity effect of amantadine HCl on bovine cornea endothelial cells, BCE C/D-1b cells were treated with various doses of amantadine HCl (0–2000 μM) for 7 days. At 24 h, the changes in cell morphology were monitored by phase-contrast microscopy. As shown in Fig. 1A, amantadine HCl did not affect the morphology of cell growth at doses ≤ 750 μM after 24 h of treatment; however, some dead cells were found that had become detached and made clusters of a small number of cells floating in the medium when cells were treated with amantadine HCl ≥ 1000 μΜ (Fig. 1A). For cell viability, MTT assay was employed for detection from days 1 to 7. Our experimental results indicated that there was no toxicity when cells were incubated with amantadine HCl at doses ≤ 20 μΜ for 7 days (Fig. 1B). After 24 h of treatment, we found that cell viability was decreased when cells were incubated with 2000 μΜ amantadine HCl (Fig. 1B). In addition, we also found that cell viability was suppressed when cells were incubated with amantadine HCl at doses ≥ 50 μΜ for three days (Fig. 1B).

### Lower doses of amantadine HCl (≤ 1000 μΜ) do not induce cell apoptosis in bovine cornea endothelial cells

**Figure 2:**
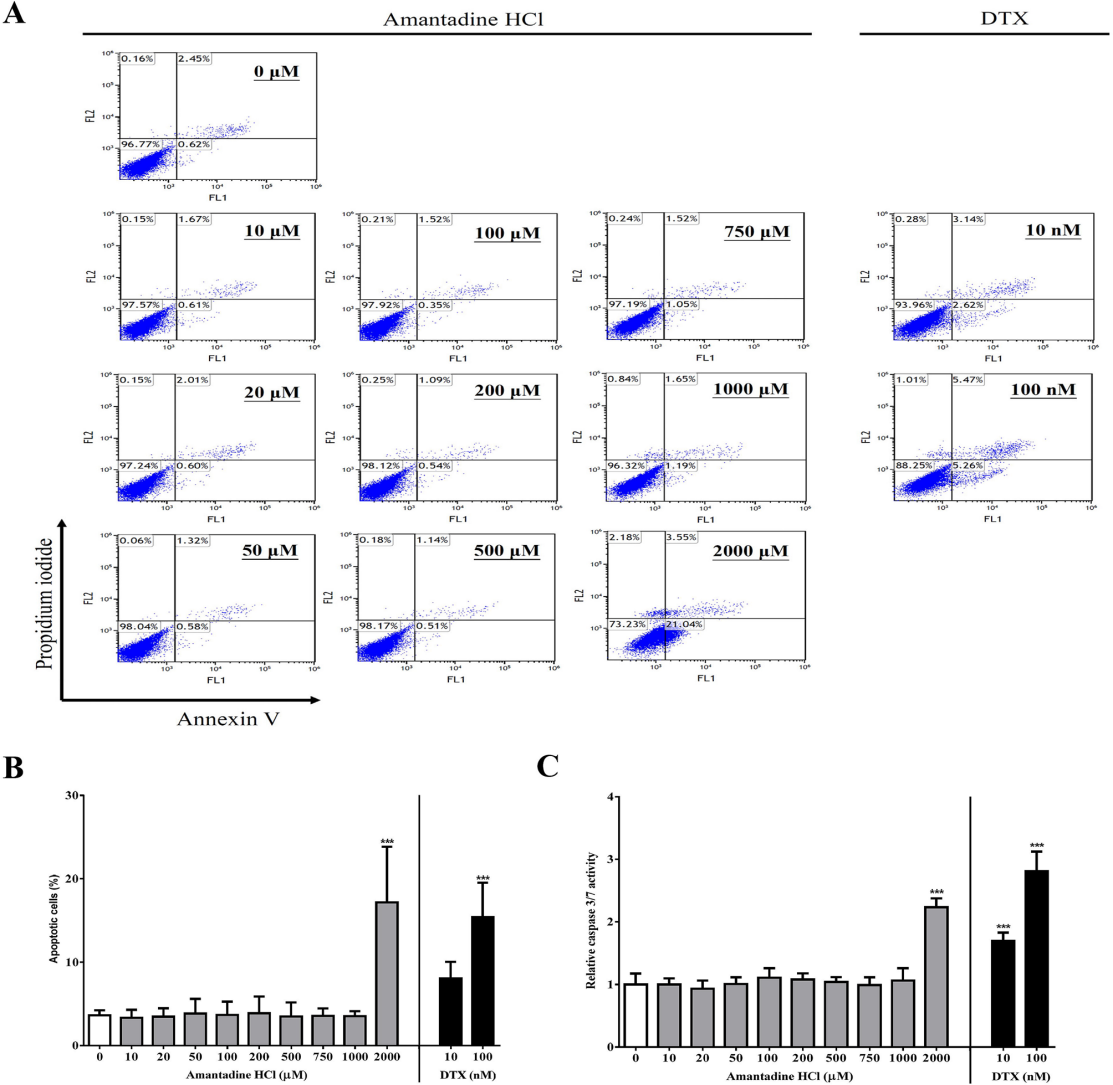
Effect of amantadine HCl on cell apoptosis of bovine cornea endothelial cells. BCE C/D-1b cells were treated with various doses of amantadine HCl (0–2000 μM) or DTX (10 and 100 nM) for 24 h. Cells were stained with Annexin V and PI and assayed by flow cytometry. (**A**) The results are representative of three independent experiments. (**B**) Statistical analysis was carried out from three independent experiments. (**C**) The activity of caspase 3/7 was analyzed by Caspase-Glo 3/7 assay kit. DTX was used as positive control. The data are presented as means ± SD of three independent experiments. Statistical significance was represented as follows: ***p < 0.001 vs. untreated control.

To examine whether amantadine HCl induces apoptosis in bovine cornea endothelial cells, BCE C/D-1b cells were treated with various doses of amantadine HCl (0–2000 μM) or docetaxel (DTX, 10 and 100 nM) for 24 h. Apoptotic cells were examined by Annexin V/propidium iodide (PI) staining and flow cytometry analysis. Experimental results indicated that lower doses of amantadine HCl (≤ 1000 μΜ) did not induce apoptosis, whereas higher dose of amantadine HCl (2000 μΜ) induced cell apoptosis in BCE C/D-1b cells (Fig. 2A,B). DTX is an anti-mitotic chemotherapeutic drug that induces cell apoptosis and arrests cell cycle progression^24^; therefore, this was used as positive control. The activity of caspase 3/7, a marker of apoptosis^25^, was also examined. BCE C/D-1b cells were treated with various doses of amantadine HCl (0–2000 μM) for 24 h. The activity of caspase 3/7 was analyzed using Caspase-Glo 3/7 assay kit. Our experimental results indicated that amantadine HCl 2000 μM significantly increased the activity of caspase 3/7, while lower doses (0–1000 μM) did not (Fig. 2C).

### Lower doses of amantadine HCl (≤ 100 μM) do not affect the progression of cell cycle, but doses of amantadine HCl at 200–1000 μM induce cell cycle arrest in G1 phase in bovine cornea endothelial cells

**Figure 3:**
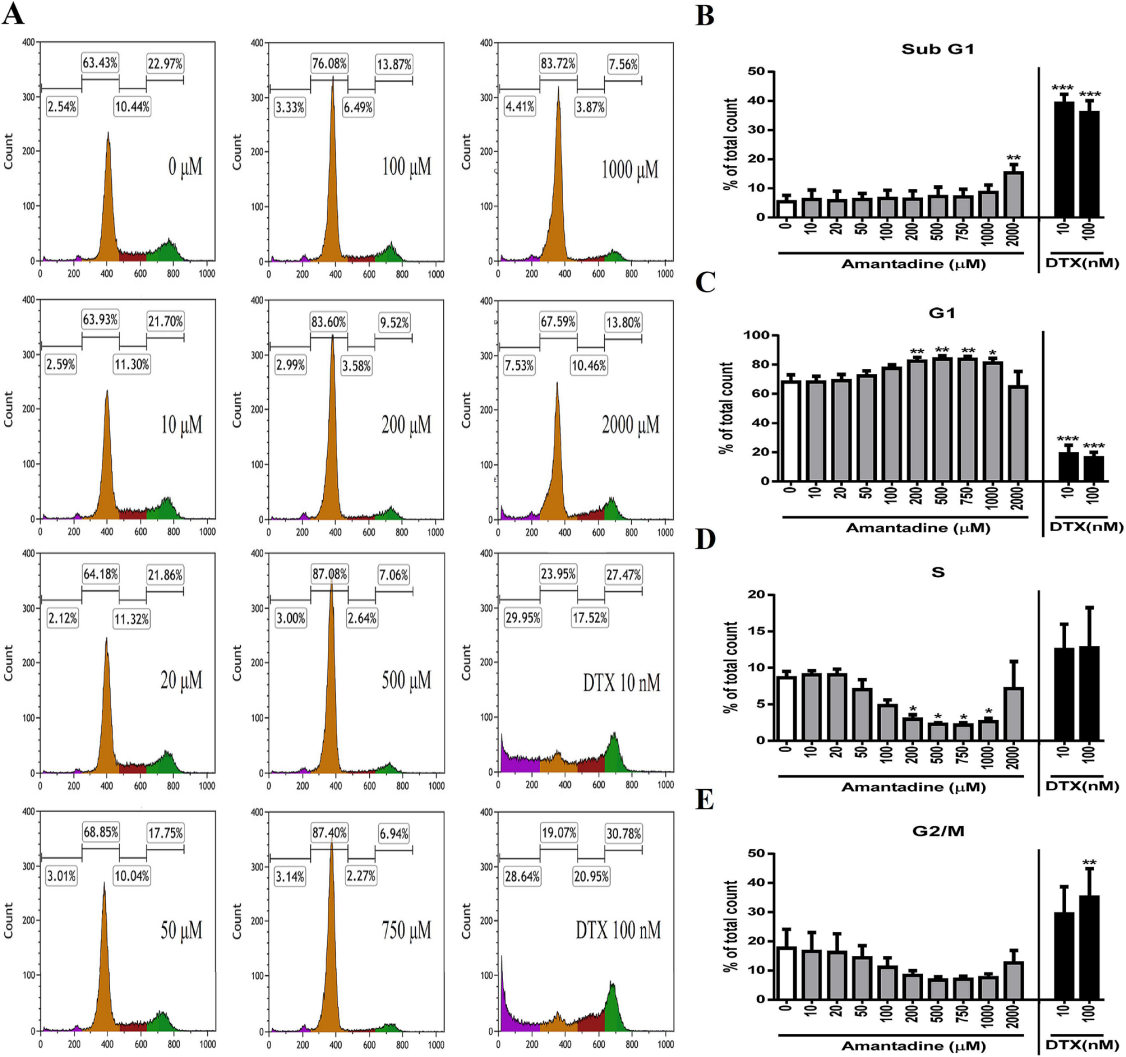
Effect of amantadine HCl on the progression of cell cycle of bovine cornea endothelial cells. BCE C/D-1b cells were treated with various concentrations of amantadine HCl (0–2000 μM) or DTX (10 and 100 nM) for 24 h. Cells were fixed, stained with PI, and then assessed by flow cytometry. (**A**) The results are representative of three independent experiments. (**B**–**E**) Statistical analysis was carried out from three independent experiments. DTX was used as positive control. The data are presented as means ± SD of three independent experiments. Statistical significance was represented as follows: *p < 0.05, **p < 0.01 or ***p < 0.001 vs. untreated control.

To examine whether amantadine HCl affected the progression of cell cycle in bovine cornea endothelial cells, BCE C/D-1b cells were treated with various doses of amantadine HCl (0–2000 μM) or DTX (10 and 100 nM) for 24 h. The progression of cell cycle was measured by flow cytometry. As shown in Fig. 3, lower doses of amantadine HCl (≤ 100 μM) did not affect the progression of cell cycle; however, experimental results indicated that doses of amantadine HCl at 200–1000 μM induced G1 arrest and decreased S proportion in BCE C/D-1b cells (Fig. 3). DTX was used as positive control.

### Doses of amantadine HCl at 0–750 μM do not cause DNA damage but attenuate cell proliferation of bovine cornea endothelial cells

**Figure 4:**
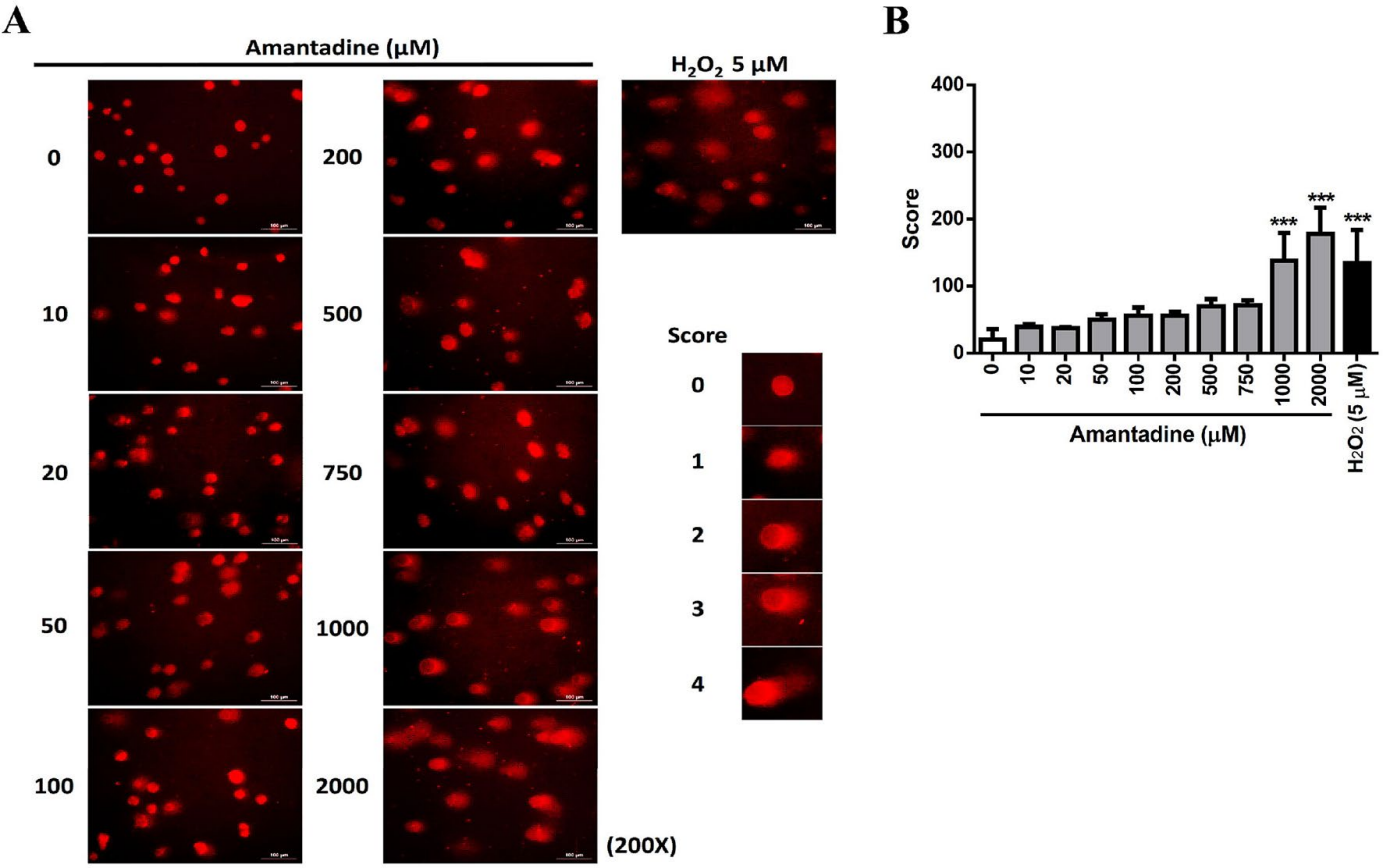
Effect of amantadine HCl on the DNA integrity in bovine cornea endothelial cells. BCE C/D-1b cells were treated with different concentrations of amantadine HCl (0–2000 μM) or H_2_O_2_ (5 μM) for 24 h. The DNA damage of cells was measured by alkaline comet assay method. One hundred cells per slide were scored into classes 0, 1, 2, 3 and 4 respectively according to the relative intensity of fluorescence in the tail. (**A**) The presentative images of three independent experiments. (**B**) Extent of DNA damage was scored and the statistical significance was represented as follows: ***p < 0.01 vs. untreated control.

**Figure 5:**
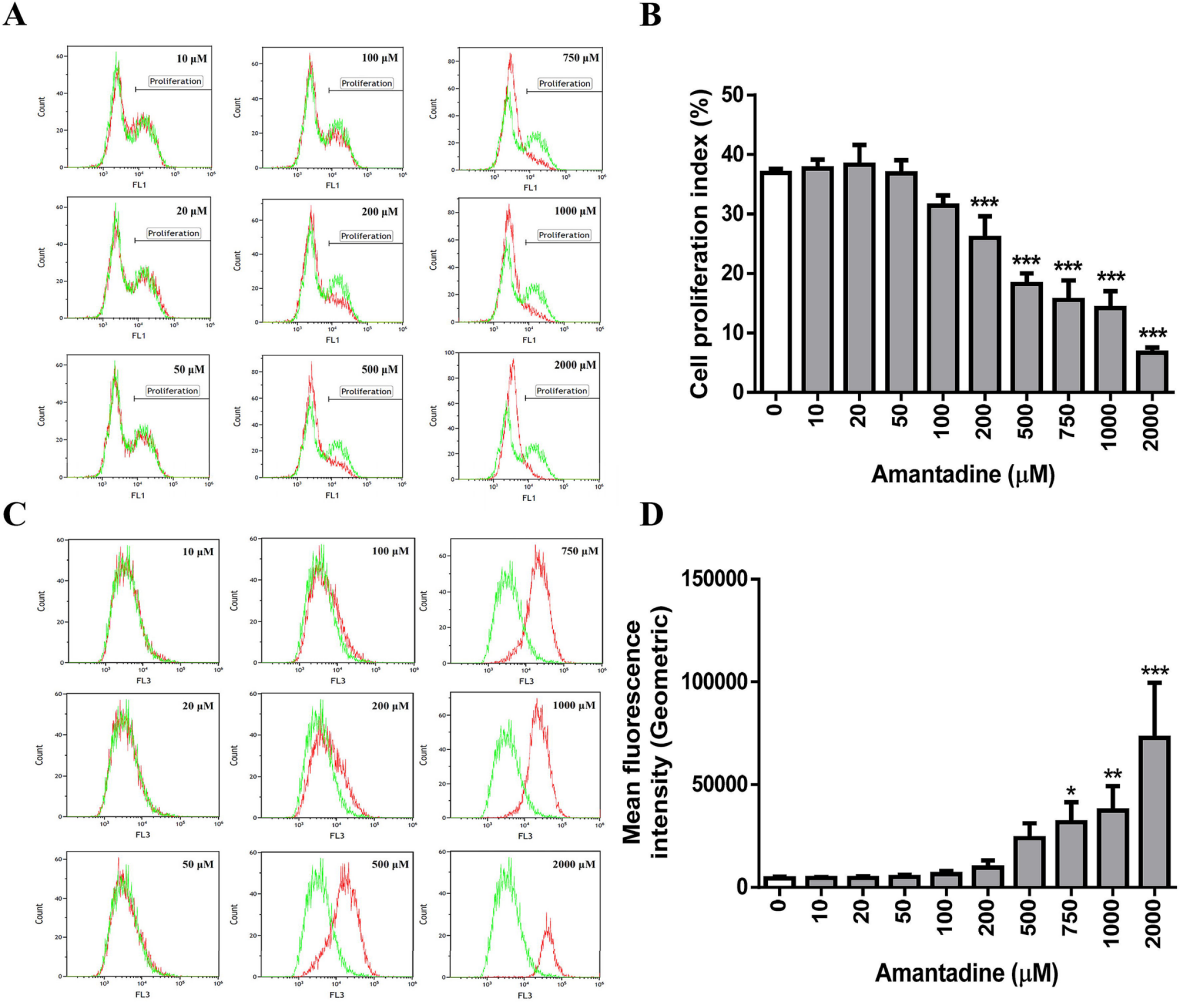
Effect of amantadine HCl on cell proliferation in bovine cornea endothelial cells. BCE C/D-1b cells were treated with different concentrations of amantadine HCl (0–2000 μM) for 24 h. (**A**) The DNA synthesis was examined by EdU incorporation assay. Overlay of the histograms of untreated cells (green) and cells treated with amantadine HCl (red). (**B**) The cell proliferation index was quantified and the statistical significance was represented as follows: **p < 0.01 vs. untreated control. BCE C/D-1b cells were labeled with 1 μΜ CFSE for 10 min and then treated with different concentrations of amantadine HCl (0–2000 μM) for seven days. (**C**) The results are representative of three independent experiments. The CFSE histograms of amantadine HCl treated cells (red) were overlaid with untreated cells (green). (**D**) Statistical analysis was carried out from three independent experiments. Data are presented as means ± SD and the statistical significance was represented as follows: *p < 0.05, **p < 0.01 and ***p < 0.001 vs. untreated control.

Since doses of amantadine HCl at 200–1000 μM were found to induce G1 arrest in BCE C/D-1b cells (Fig. 3), these doses of amantadine HCl were further examined to assess whether they affected DNA integrity, DNA synthesis and cell proliferation. To test the effect of amantadine HCl on DNA damage, the alkaline comet assay was employed to detect the single-strand DNA breaks. As shown in Fig. 4, doses of amantadine HCl lower than 1000 μM (0–750 μM) had no significant effect on DNA damage vis-à-vis higher doses of amantadine HCl (1000–2000 μM). H_2_O_2_ was used as positive control since it is a source of ROS which can cause DNA damage^26^. For the DNA synthesis, experimental results indicated that amantadine HCl significantly inhibited the EdU incorporation at doses ≥ 200 μΜ (Fig. 5A,B). In addition, the results of CFSE cell proliferation assay showed that amantadine HCl attenuated cell proliferation at doses ≥ 750 μΜ (Fig. 5C,D).

### Higher doses of amantadine HCl (≥ 1000 μM) induce endothelial hyperpermeability

**Figure 6:**
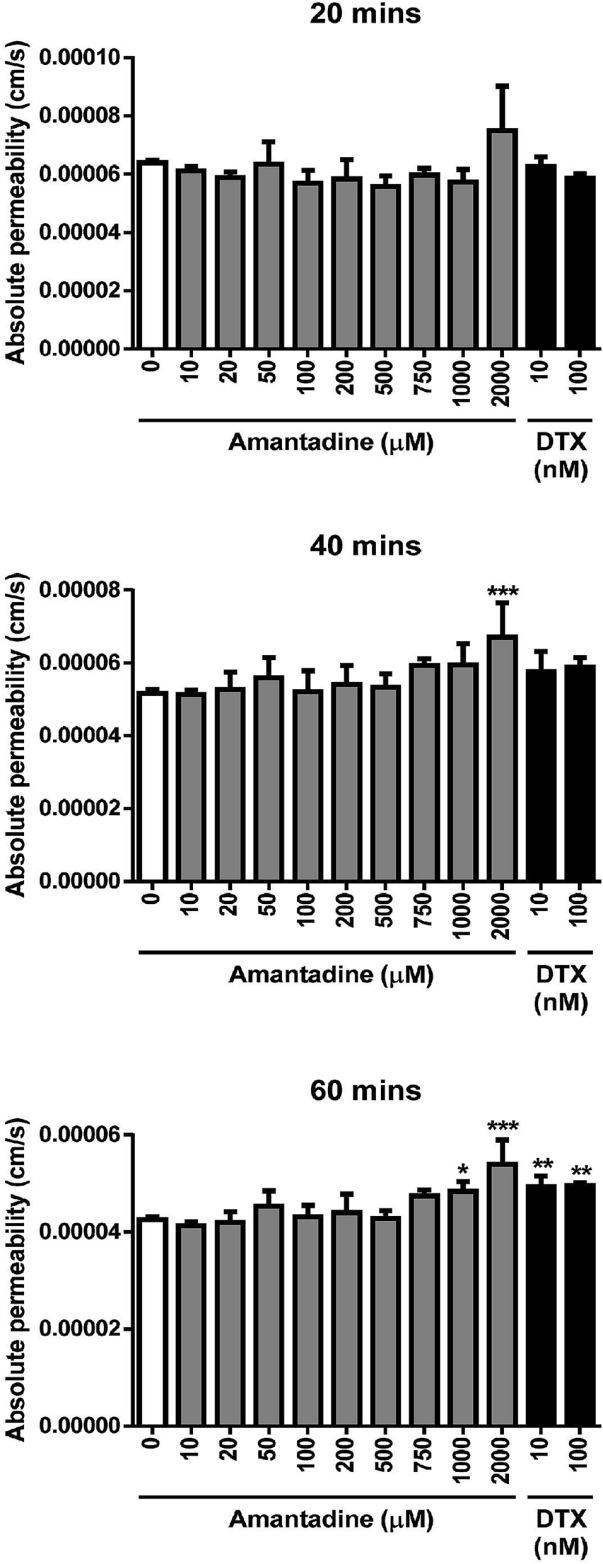
Effect of amantadine HCl on endothelial permeability in bovine cornea endothelial cells. BCE C/D-1b cells were seeded in the upper chamber of 0.4 μm transwell inserts and treated with different concentrations of amantadine HCl (0–2000 μM) or DTX (10 and 100 nM) for 24 h. Afterward, FITC-dextran (1 mg/mL) was added into the upper chamber, then the lower chamber media was collected after 0, 20, 40 or 60 min, and fluorescent intensity was measured (ex: 485 nm; em: 535 nm) using a fluorescence plate reader. The absolute permeability was presented as means ± SD of three independent experiments. The statistical significance was represented as follows: *p < 0.05, **p < 0.01 and ***p < 0.001 vs. untreated control.

Endothelium maintains stromal deturgescence through barrier and pump functions. While the barrier function limits excessive fluid influx into the stroma from the anterior chamber, the fluid pump function counterbalances fluid leaks through the paracellular space^27^. Altered endothelial cell function and abnormalities or damage in the endothelial cell barrier might lead to corneal edema. To further examine whether amantadine HCl affects endothelial permeability, in vitro endothelial permeability was performed and the passage of FITC-dextran was examined. Experimental results indicated that doses of amantadine HCl ≤ 750 μM had no significant effect on the permeability of FITC-dextran at 1 h compared to untreated control; however, higher doses of amantadine HCl (≥ 1000 μM) significantly increased cell permeability, indicating that ≥ 1000 μM amantadine might lead to damage of the endothelial cell barrier (Fig. 6). DTX was used as positive control.

## Discussion

Unlike bovine corneal endothelial cells^28^, human corneal endothelial cells do not regenerate in vivo and exhibit limited proliferative capability in vitro caused by contact-inhibited growth arrest at the G1 phase^29^. In addition, human corneal endothelial cells are not easy to obtain; therefore, in the present study, we tested the cytotoxic effect of amantadine HCl using bovine corneal endothelial cells. Our experimental results showed that acute toxicity of amantadine HCl on bovine cornea endothelial cells was not observed at doses ≤ 100 µM for 24 h; however, it was found that doses of amantadine HCl ≥ 200 µM induced cell cycle arrest at G1 phase and resulted in the inhibition of both DNA synthesis and cell proliferation.

A recent study indicated that incubation of bovine corneal samples with 200 μM amantadine HCl for 6 h did not increase cell death compared with untreated control samples, but cell height was significantly increased compared to controls, which might result in cell death and reduce the density of corneal endothelial cells noted in patients on amantadine HCl therapy^30^. Notably, the experimental results indicated that higher doses of amantadine HCl (≥ 1000 μΜ) had significantly cytotoxicity with consistently toxic effects on bovine cornea endothelial cells including inhibiting cell growth and proliferation, inducing DNA damage and apoptosis, and increasing endothelial permeability. A previous study indicated that amantadine was transported principally across the blood–brain barrier by a saturable transport system with a one-half saturation concentration of about 1.0 mM^31^. The mean amantadine concentrations in human brain tissue ranged from 48.2 to 386 μM when the duration of treatment was ≥ 10 days and the drug-free time ≤ 3 days^12^. Corneal endothelium has been thought to be nonmitotic cells that have no potential in regeneration and reparation^32–34^. Accumulated doses of amantadine HCl might cause cornea edema. In addition, a nationwide cohort study of patients with PD in Taiwan indicated that amantadine HCl increases the risk of cornea edema in a concentration-dependent manner (a hazard ratio of 2.05 for a moderate dose (2000–4000 mg) and 2.84 for a high dose (4000 mg)^11^; therefore, to judge the toxic effect of amantadine HCl, the exact concentration of amantadine HCl in the cornea should be further examined.

Previous studies pointed out that the CTG18.1 repeat expansion might reduce *TCF4* gene expression^35^ and Hessen et al. examined the copy number of CTG18.1 trinucleotide repeat in the *TCF4* gene by an amantadine HCl-associated corneal edema patient^36^. Although they did not find the change on the copy number of CTG18.1 trinucleotide repeat in the *TCF4* gene, genetic variation remains an important issue that might create sensitivity to amantadine HCl treatment, leading to corneal edema. In the present study, although the cytotoxic dosages of amantadine HCl were much higher than in clinical situations, accumulated doses of amantadine HCl might still pose risk to cause corneal edema in patients with rare genetic variations.

There are several limitations in the present study. Firstly, because human cornea endothelial cells are not easy to obtain, bovine cornea endothelial cells were used to examine the toxic effect of amantadine HCl, and there might well be differences between bovine and human cornea endothelial cells. Secondly, further experiments could not proceed due to the lack of antibodies to bovine cells; thirdly, an in vitro experimental model could not achieve the cumulative dose of amantadine HCl in vivo; fourthly, the in vitro cytotoxic assays used in this study could not fully represent the real saturation of corneal edema; and finally, the duration of amantadine treatment in the present study might not represent the effects of the drug in real life due to edema formation in a physiological sense manifesting over time.

To our knowledge, this is the first study to successfully examine the cytotoxic effects of amantadine HCl using cornea endothelial cells, having performed the evaluation of these on cell growth, proliferation, apoptosis, and endothelial permeability as well as DNA integrity in bovine cornea endothelial cells. Our experimental results indicated that no cytotoxic effect of amantadine HCl on bovine cornea endothelial cells was observed at doses ≤ 100 µM for 24 h. However, doses of amantadine HCl ≥ 200 µM induced cell cycle arrest at G1 phase and resulted in the inhibition of both DNA synthesis and cell proliferation in bovine cornea endothelial cells after 24-h treatment. Doses of amantadine HCl ≥ 1000 μΜ had cytotoxic effects on bovine cornea endothelial cells including inhibiting cell growth and proliferation, inducing DNA damage and apoptosis, and increasing endothelial permeability after 24-h treatment. In a 72-h treatment, doses of amantadine HCl ≥ 50 μΜ attenuated cell growth on bovine cornea endothelial cells. The cytotoxic dosages of amantadine HCl on bovine cornea endothelial cells might provide a hint for further evaluating the toxic effect of amantadine on corneal edema.

## Material and methods

### Reagents

Dulbecco’s modified Eagle's medium (DMEM), penicillin and streptomycin were obtained from Corning Cellgro (Manassas, VA, USA). Fetal bovine serum (FBS) was obtained from Gibco-BRL (Life Technologies, Grand Island, NY, USA). Amantadine HCl (A1260; Molecular Weight: 187.7), 3-(4,5-dimethylthiazol-2-yl)-2,5-diphenyltetrazolium bromide (MTT), PI, Triton X-100, ribonuclease A (RNase A), DTX, normal melting point agarose, low melting point agarose and fluorescein isothiocyanate (FITC)-dextran (40 kDa) were obtained from Sigma (St. Louis, MO, USA). Caspase-Glo 3/7 assay kit was purchased from Promega (Madison, WI, USA). Alexa Fluor 488 Annexin V/Dead Cell Apoptosis kit and Click-iT EdU Alexa Fluor 488 flow cytometry assay kit were purchased from Thermo Fisher Scientific (Waltham, MA, USA). CFSE cell-division tracker kit was obtained from BioLegend (San Diego, CA, USA).

### Cell culture

The bovine cornea endothelial cell line, BCE C/D-1b cell, was purchased from American Type Culture Collection (CRL-2048, Manassas, VA, USA). BCE C/D-1b cells were cultured in DMEM supplemented with 10% heat-inactivated FBS, 100 U/mL penicillin and 100 U/mL streptomycin in a humidified atmosphere of 5% CO_2_ at 37 °C. The cells were used for experiments at passages 6–20. Amantadine HCl was dissolved in PBS to prepare a 50 mg/mL (266.3 mM) stock solution and stored at − 20 °C. For the preparation of working solution, amantadine HCl was diluted into 10 mM using complete DMEM medium. The osmolarity of the incubation media with amantadine HCl treatments (0–2000 μM) was detected by micro-osmometer (Model 210, Fiske, Norwood, MA, USA) and the results showed that all incubation media were isotonic solutions (330 ± 2 mOsm/kg).

### Cell viability assay

Cell viability was examined by the MTT colorimetric assay. A total of 1 × 10^4^ cells was seeded in 96‑well plates and cultured overnight for attachment. Various concentrations of amantadine HCl (0–2000 μM) were treated for 1 to 7 days. Half of the cultured medium was replaced with fresh amantadine HCl every two days. Thereafter, 0.1 mg MTT was dissolved in DPBS and then added into each well and incubated for 4 h at 37 °C. Afterward, the formed formazan crystals were solubilized using 100 µL hydrochloric acid–isopropanol (1 portion of 4 N HCl: 100 portion of isopropanol). After 20 min of solubilization, the absorbance of 570 nm was measured with a microplate reader (BioTek Instruments, Winooski, VT, USA).

### Apoptosis assay

The apoptotic cells were detected using Annexin V and PI staining and the method was modified from a previous study^37^. Briefly, BCE C/D-1b cells were treated with different doses of amantadine HCl (0–2000 μM) for 24 h. Cells were stained with Alexa Fluor 488 Annexin V and PI in binding buffer according to the manufacturer’s protocol (Thermo Fisher Scientific, Waltham, MA, USA) and analyzed by FC500 flow cytometer (Beckman-Coulter, Fullerton, CA, USA). A total of ten thousand events were collected per sample, and data were acquired and processed using CXP analysis software (Beckman-Coulter, Fullerton, CA, USA).

### Caspase 3/7 activity assay

The caspase 3/7 activity assay was used to detect the activity of caspase 3 or 7 in the cells and the method was modified from a previous study^37^. Briefly, a total of 5 × 10^3^ BCE C/D-1b cells were seeded in a 96-well white plate and allowed to acclimatize overnight. Afterward, cells were treated with different doses of amantadine HCl (0–2000 μM) for 24 h. Thereafter, Caspase-Glo 3/7 reagent was added to each well and gently mixed using a plate shaker at 300–500 rpm for 30 s, and then samples were incubated for 30 min at RT. Enzyme activity was directly proportional to luminescence. The luminescence intensity was detected by a luminescence microplate reader (BioTek Instruments, Winooski, VT, USA), and the data were normalized relative to the caspase 3/7 activity of cells treated with DMSO alone.

### Cell cycle analysis

The cell cycle analysis was detected DNA content using flow cytometry and the method was modified from a previous study^38^. Briefly, BCE C/D-1b cells were treated with different doses of amantadine HCl (0–2000 μM) for 24 h. Cells were trypsinized, washed twice by cold PBS, and fixed in 70% ethanol overnight at 4 °C. After fixation, cells were washed twice with PBS and then incubated in PBS containing PI, RNase A and Triton X-100 at 4 °C for 30 min. The cell cycle phase distribution was assessed by FC500 flow cytometer (Beckman-Coulter, Fullerton, CA, USA). Data were analyzed by Kaluza analysis software (Beckman Coulter, Brea, CA, USA).

### Comet assay

The alkaline comet assay was used for the detection of DNA single-strand breaks and performed according to our previous study^39^. Briefly, plain glass slides were pre-covered with 1% normal melting point agarose in PBS (pH 7.4) and allowed to dry on a flat surface at room temperature. BCE C/D-1b cells were treated with different doses of amantadine HCl (0–2000 μM) for 24 h. A total of 10^5^ cells were gently mixed with 0.5% low melting point agarose in PBS (pH 7.4), rapidly layered onto the precoated slides, and covered with a coverslip. After removing the cover slip, cells were immersed in a freshly made alkaline lysis solution (2.5 M NaCl, 100 mM Na_2_EDTA, 10 mM Tris and 1% Triton X-100 at pH 10) at 4 °C for 1 h. Afterward, the slides were placed in an electrophoresis tank containing 0.3 M NaOH and 1 mM Na_2_EDTA and run electrophoresis (30 V, 300 mA) for 15 min at 4 °C. Slides were soaked in a cold neutralizing buffer (400 mM Tris buffer, pH 7.0) at 4 °C for 5 min, stained with PI (2.5 μg/mL), and analyzed by fluorescence microscopy. One hundred cells per slide were scored into classes 0, 1, 2, 3 and 4 respectively according to the relative intensity of fluorescence in the tail.

### Cell proliferation assay

For cell proliferation, this was evaluated at two levels: DNA synthesis and cell division using EdU incorporation assay and CFSE cell-division tracking respectively. For the detection of DNA synthesis, BCE C/D-1b cells were treated with different doses of amantadine HCl (0–2000 μM) for 24 h and then 5 μM EdU was added into the cell culture medium for 6 h. The cellular EdU content was measured by flow cytometry. For the detection of cell division, BCE C/D-1b cells were labeled with 1 μΜ CFSE for 10 min at 37 °C and protected from light. Cells were washed three times with DMEM containing 10% FBS and then treated with different doses of amantadine HCl (0–2000 μM) for 7 days. The dividing cells were detected by flow cytometry.

### In vitro permeability assay

Cell permeability assay was modified according to a previous study^40^. Briefly, 12-transwell inserts (0.4 μm polyester membrane, Corning, New York, USA) were coated with collagen type I (5 μg/cm^2^, Corning, New York, USA) at room temperature for 30 min and then incubated at 37 °C for 2 h. A total of 2.5 × 10^4^ BCE C/D-1b cells were seeded into the inserts and allowed to acclimatize overnight. Cells were treated with various doses of amantadine HCl (0–2000 μM) or DTX (10 and 100 nM) for 24 h and then treated with 1 mg/mL FITC-dextran on the upper chamber. 100 μL samples were taken after 0, 20, 40 or 60 min respectively from the lower chamber, and fluorescent intensity was measured (ex: 485 nm; em: 535 nm) using a fluorescence plate reader (Epoch, Biotek Instruments, USA). The removed volume was replaced by fresh medium. The absolute permeability P [cm/s] was calculated by the following equation.

$$P = \left[ {{\text{C}}\left( {\text{t}} \right) - {\text{C}}\left( {{\text{t}}0} \right)} \right] \cdot {\text{V}})/\left( {{\text{A}} \cdot {\text{t}} \cdot {\text{C}}0} \right)$$

C(t): FITC-dextran concentration (μg/mL) at time point selected for calculation; C(t0): FITC-dextran concentration (μg/mL) at 0 min; V: volume (cm^3^) in the lower chamber; A: surface of transwell membrane (cm^2^); t: duration of the flux (s); C0: initial FITC-dextran concentration (μg/mL) in the upper chamber.

There are several limitations in the present study. Firstly, because human cornea endothelial cells are not easy to obtain, bovine cornea endothelial cells were used to examine the toxic effect of amantadine HCl, and there might well be differences between bovine and human cornea endothelial cells. Secondly, further experiments could not proceed due to the lack of antibodies to bovine cells; thirdly, an in vitro experimental model could not achieve the cumulative dose of amantadine HCl in vivo; fourthly, the in vitro cytotoxic assays used in this study could not fully represent the real saturation of corneal edema; and finally, the duration of amantadine treatment in the present study might not represent the effects of the drug in real life due to edema formation in a physiological sense manifesting over time.

### Statistical analysis

All data are expressed as means ± SD. Each value is the mean of three independent experiments. Statistical analysis was assessed via one-way ANOVA followed by Tukey post-hoc test using IBM SPSS Statistics v.19 (IBM Corp., Armonk, NY, USA), and the significant difference was set at *p < 0.05; **p < 0.01; ***p < 0.001.
